# Case report of worsening of preexisting pericardial effusion after ASD device closure: is it calamitous?

**DOI:** 10.1186/s43044-021-00171-8

**Published:** 2021-06-05

**Authors:** Zahra Khajali, Ata Firouzi, Hamidreza Pouraliakbar, Zahra Hosseini, Fateme Jorfi

**Affiliations:** 1 Shaheed Rajaei Cardiovascular Medical and Research Center, Tehran, Iran; 2 Atherosclerosis Research Center, Ahvaz Jundishapur University of Medical Sciences, Ahvaz, Iran

**Keywords:** Atrial septal defects, Congenital heart defect, Septal occluder device, Pericardial effusion, Echocardiography

## Abstract

**Background:**

Secundum-type atrial septal defects (ASD) constitute 8% to 10% of congenital heart defect. Secundum ASDs can be closed either percutaneously or surgically. However, ASD device closure has proven to be technically safe and feasible; it is not free of complications. These complications include device embolization/malposition which have been reported in 3.5% of cases, arrhythmia, and pericardial effusion in 2.6% and 0.5–1.5% respectively, device thrombus, residual shunting, and impingement of the device on the adjacent structures.

**Case presentation:**

We introduce three patients with secundum ASD who had preexisting pericardial effusion, device closure was performed for them, and after the procedure, the effusion size progressed significantly. We used multimodality imaging to diagnose the cause of pericardial effusion (PE). Cardiac erosion was diagnosed in one of the patients that managed surgically. We did not found any specific procedure-related cause for worsening the pericardial effusion in the other two patients.

**Conclusion:**

Several reasons include procedure-related complication and other systemic causes should be considered in patients who develop pericardial effusion after trans catheter closure of ASDs.

## Background

Secundum-type atrial septal defects (ASDs) constitute 8% to 10% of congenital heart defect [1]. Secundum ASDs can be closed either percutaneously or surgically. In 2020, thanks to the better appreciation, the pathophysiology of cardiovascular diseases continued advances in engineering in interventional equipment, miniaturization of materials, and improvements in knowledge of interventional techniques; percutaneous interventions in complex cardiovascular diseases are growing up. New image-guided procedures and high-precision imaging technology have provided feasible complex interventions for clinicians.

Trans catheter closure (TCC) has dramatically improved the management of ASDs in such a way that TCC is being the method of choice for closing the defects in eligible ones [2]. ASD device closure is not free of complications. These complications include device embolization/malposition which have been reported in 3.5% of cases, arrhythmia and pericardial effusion in 2.6% and 0.5–1.5% respectively, device thrombus, residual shunting, and impingement of the device on the adjacent structures [3, 4].

Pericardial effusion after device closure is one of the ASD closure complication that could be resolved spontaneously, but it might be a sign of a catastrophic complication; it means cardiac erosion. Erosions in adjacent cardiac structures are very rare (0.2–0.3% of cases) after TCC of ASDs and in most cases happen within the first 6 months after ASD occluder implantation [5].

The true diagnosis for an appropriate management is a prime issue in those with post TCC of ASDs with pericardial effusion. By the way, we also have cases with preexisting PE whom the amount of the effusion has gotten worse after the procedure.

In this paper we introduce three patients with secundum ASD who had preexisting pericardial effusion and device closure was performed for them and after the procedure, the effusion size progressed significantly. We discuss about the individualized approach and management of these patients.

## Case presentation

### Case 1

**Fig. 1:**
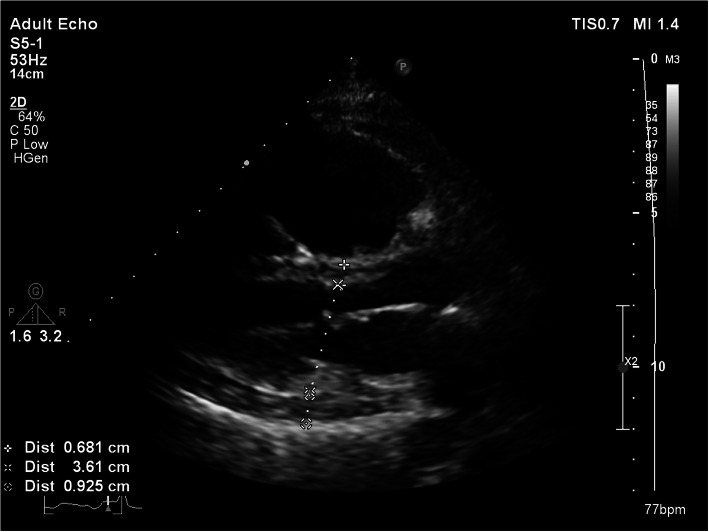
Parasternal long axis TTE view showed mild pericardial effusion in the first patient

A 55-year-old female patient, a known case of end-stage renal disease (ESRD) on hemodialysis, was referred to our hospital after being diagnosed with a 20-mm secundum ASD in pre-operative evaluation for renal transplantation. In transesophageal echocardiography (TEE), moderate right ventricle enlargement and pulmonary hypertension were detected. Pre-procedural TEE showed: Normal left ventricle (LV) systolic function (LVEF: 55%) with LV diastolic dysfunction grade I, moderate right ventricle (RV) enlargement, and mild systolic dysfunction. A large secundum ASD with a significant left to right shunt with sufficient rims for device closure was notable. Moderate tricuspid regurgitation (TR) with tricuspid regurgitant gradient (TRG), 40 mmHg, and moderate pulmonary arterial hypertension (PAH) were reported. Mild circumferential pericardial effusion (PE) was also demonstrated (Fig. 1). The patient was scheduled for right heart catheterization and transcatheter ASD closure. Under TEE guidance, after 15 min balloon occlusion test, left ventricular end-diastolic pressure (LVEDP) was about 20 mmHg; so, based on stop flow diameter (SFD: 20 mm) in TEE, ASD closure was performed with a 24-mm fenestrated ASD-6 mm (FASD) occluder device (Occlutech) due to persistent elevated LV end-diastolic pressure after sizing balloon occlusion test and also elevated pulmonary vascular resistance (PVR).

**Fig. 2:**
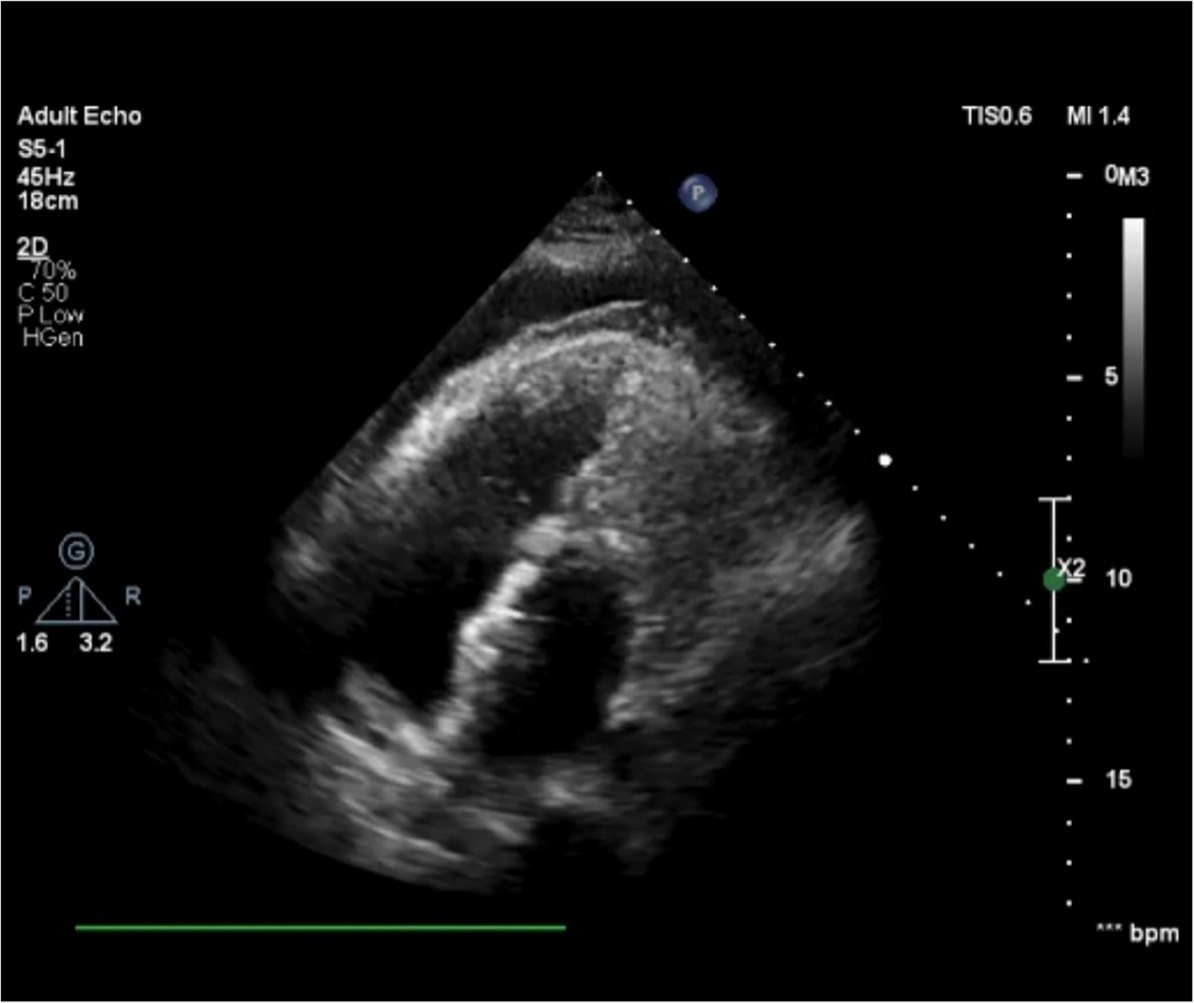
post TTC of ASD in the first patient TTE revealed moderate to severe PE

**Fig. 3:**
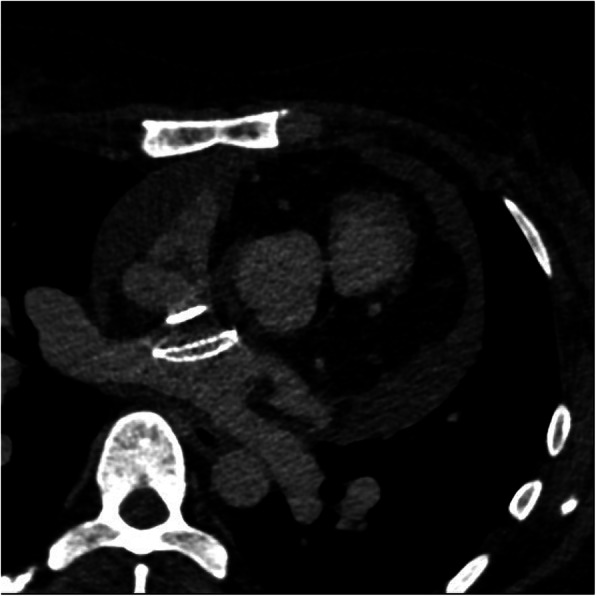
CT angiography showed that ASO device was in proper position through the interatrial septum and there was a retroaortic hematoma due to cardiac erosion

TTE on the first day after the procedure revealed moderate to severe PE (Fig. 2). Spiral chest CT angiography (CTA) illustrated an erosion of the left atrium (LA) roof and hematoma around the aorta. Thus, she was transferred to the operation room immediately and the surgeon confirmed the diagnosis of LA erosion (Fig. 3). The device was removed and the ASD was repaired with a patch.

### Case 2

**Fig. 4:**
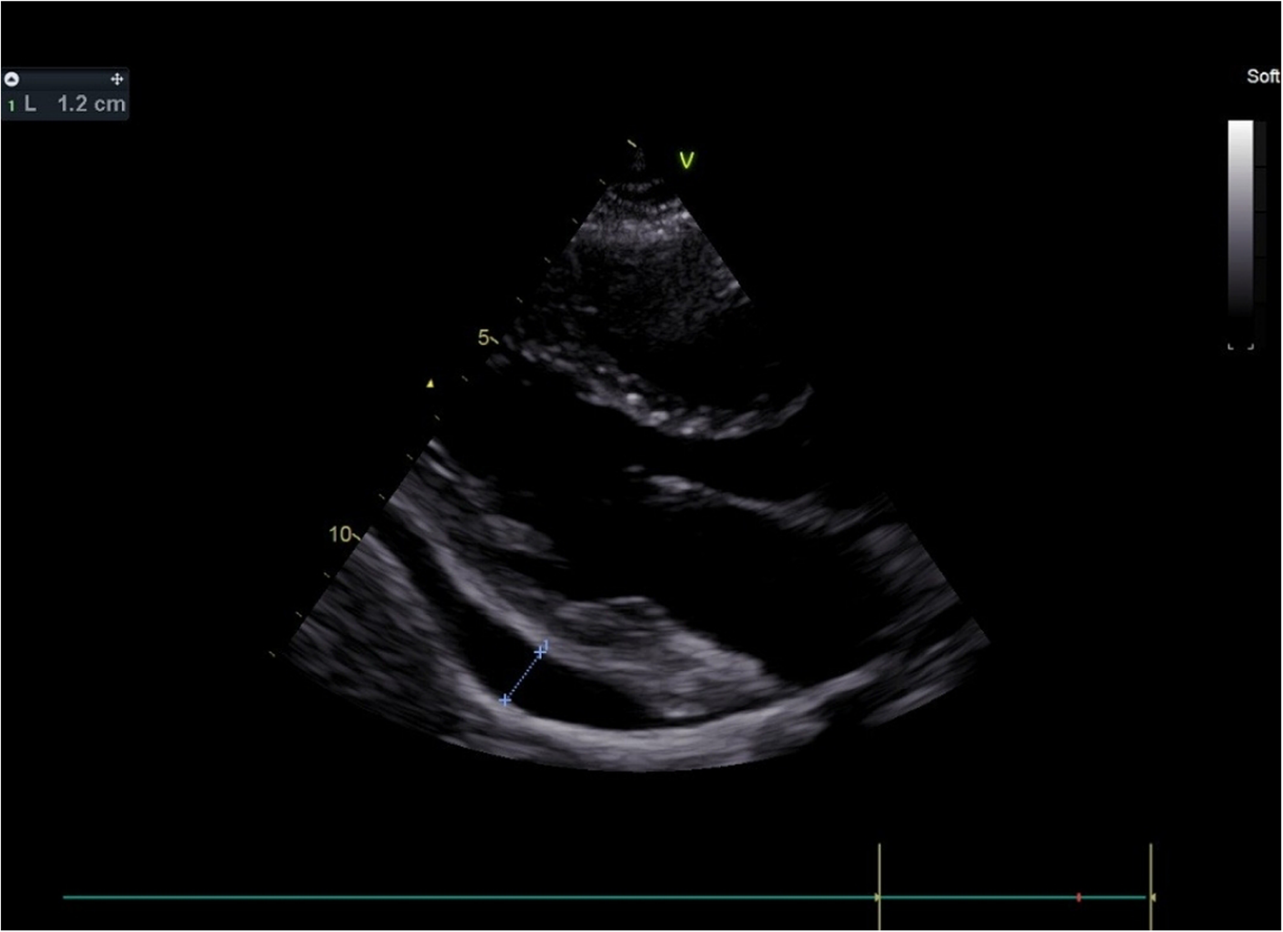
Mild to moderate PE in the second patient was detected by TTE

A 34-year-old woman presented to our hospital with complaints of exertional dyspnea (New York Heart Association [NYHA] functional class II) who in TTE had mild to moderate RV enlargement and dysfunction with ASD secundum and mild to moderate circumferential PE (Fig. 4). We followed her for 2 to 3 months, and she was on colchicine with no change in the amount of pericardial effusion; so, we decided to close the ASD. During heart catheterization, due to the patient’s young age and lack of evidence of left ventricular disease, a balloon occlusion test was not performed for her, and we found that the patient had actually two separated ASDs which were closed under TEE guidance with two ASO devices successfully without obvious complication during the procedure. The next day, the patient was symptomatic (sinus tachycardia) and TTE revealed that the severity of pericardial effusion was dramatically increased. Spiral chest CTA showed no evidence of cardiac erosion or hematoma. She underwent pericardiocentesis and clear fluid was drained. After that, the patient was hemodynamically stable and discharged without any pericardial effusion.

### Case 3

**Fig. 5:**
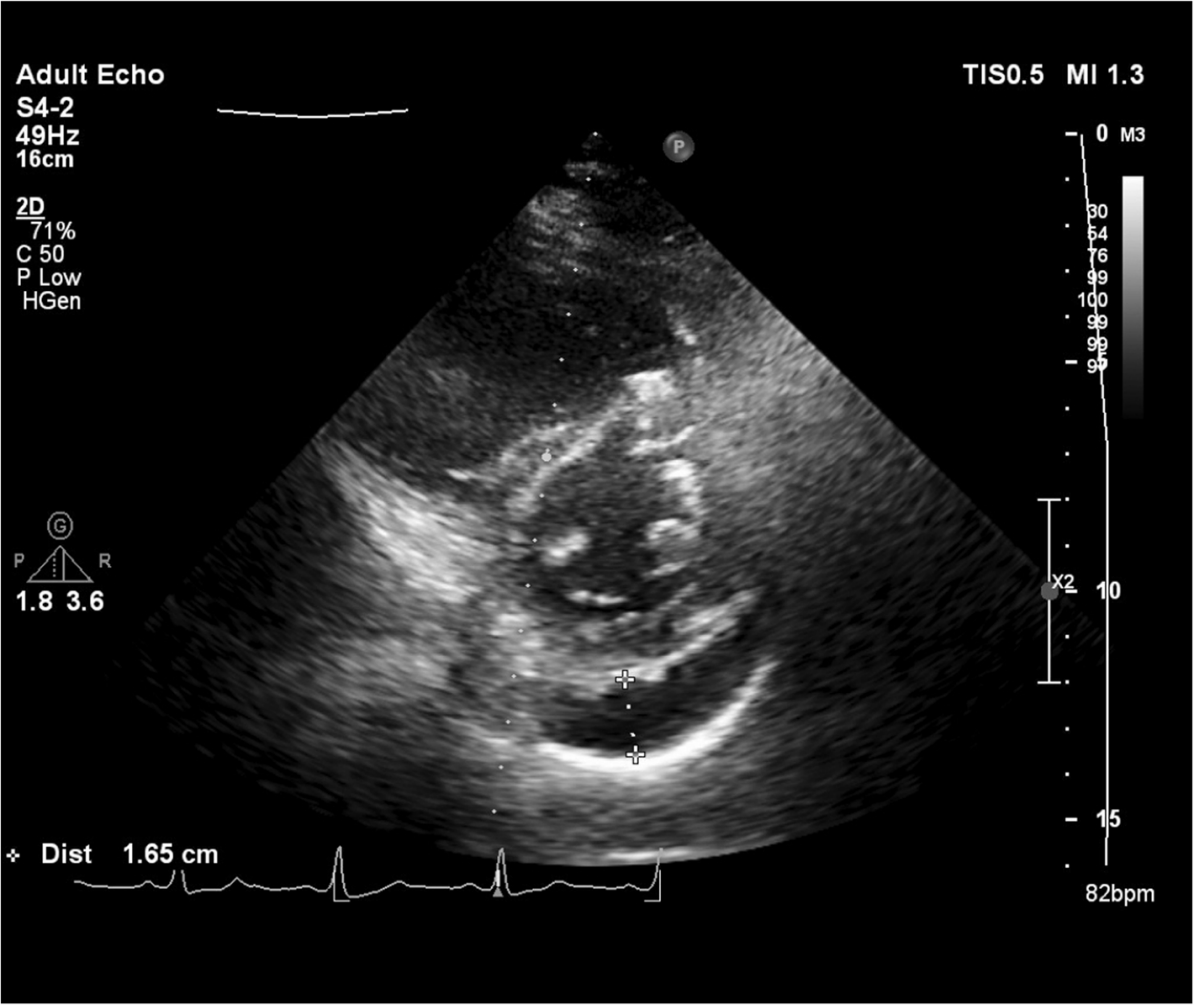
Increased PE in the third patient after ASO implantation was seen in parasternal long axis TTE view

**Fig. 6:**
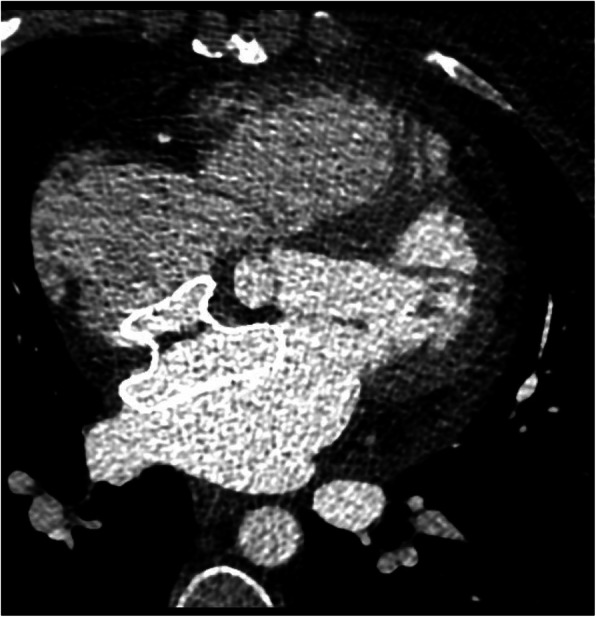
CTA showed no evidence of cardiac erosion or perforation

A 60-year-old woman with a feeling of palpitation and exertional dyspnea was referred to our center with a diagnosis of secundum ASD for closure. Preprocedural TEE demonstrated normal LV size and function, moderate RV dilation and mild dysfunction, moderate ASD secundum size with suitable surrounding rims, and mild to moderate PE. She had left ventricle end-diastolic pressure of 10 mmHg before and after balloon occlusion test on heart catheterization, so she underwent transcatheter ASD closure via Occlutech septal occludder 21 mm device with no residual shunt. Six hours after the procedure, pericardial effusion increased in size without any symptoms or hemodynamic disturbance on TTE (Fig. 5). In CTA, no evidence of erosion or hematoma was detected (Fig. 6). Steroid and colchicine were started for her, and after a couple of days, she was discharged with good conditions. During follow-up echocardiography after 3 months, mild PE was reported.

## Discussion

The most common atrial septal defect occurs in the central part of the atrial septum in the region of fossa ovalis called secundum ASD [1]. Most patients with ASD are asymptomatic and may remain undiagnosed until later in life. In adults worsening of the clinical condition has been attributed to various factors such as an increased LV end-diastolic pressure and reduced LV compliance, RV failure, atrial arrhythmia, and elevated pulmonary artery pressure [1].

Secundum ASDs can be closed surgically or with percutaneous devices. The first device closure of secundum ASD was done by Mills and King in 1976 [6]. Percutaneous ASD closure has become a popular procedure in defects with suitable size and septal rims. Common complications associated with transcatheter ASD closure include residual shunts, embolization, device-related thrombosis, erosion and perforation of the heart, infective endocarditis, and sudden death [7].

Post ASD closure pericardial effusion occurs with a reported incidence of 0.5–1.5 % secondary to cardiac perforation, device erosion, or reactive PE. Pre-existing PE could be due to different causes include uremic, autoimmune, hypothyroidism, Viral, bacterial, fungal or parasitic infections. In a retrospective study (*n* = 40), Spodick et al. found 13 of their 40 patients (32.5%); > 21 years of age with isolated secundum ASDs have circumferential PE preoperatively [8].

Our first patient had mild pre-existing PE secondary to uremia that aggravated after device implantation. The first modality for determining the etiology of the worsening of PE is Spiral Cardiac CT Angiography (CTA) which in this patient confirmed the disastrous complication, it means cardiac erosion; so, immediate surgical management of the patient was performed.

The second patient had mild to moderate pre-existing PE with no response to anti-inflammatory agents during 2- to 3-month follow-up that seems to be due to ASD itself. With mild to moderate pre-existing PE, Reddy et al. speculated that, if septal defects were left untreated, the effusion may accumulate over time because of chronic volume overload, which may lead to more PE [9]. The amount of PE in this patient also increased significantly after two ASD device implantations that necessitate pericardiocentesis. Cardiac CTA revealed no evidence of cardiac perforation, device erosion, or cardiac hematoma. Reactive PE was assumed as an etiologic factor that worsened the patient’s pre-existing PE.

The third patient had mild to moderate PE due to unknown etiology that after device implantation increased to moderate PE without evidence of cardiac perforation, erosion, or hematoma, so we managed her conservatively. Fortunately, she was asymptomatic and the PE decreased significantly within 3-month follow-up.

Generally speaking, several reasons should be considered in patients who develop pericardial effusion after TCC of ASDs; the most and catastrophic cause is cardiac erosion which frequently happens at the left atrium roof or aortic side. So, the immediate diagnostic modality which could rule out is cardiac CTA; if no evidence of cardiac erosion was found, looking for hematoma around the pulmonary vein or left atrium appendage which commonly occurs by wire manipulation which leads to perforation should be appraised. In fact, the diagnosis of cardiac erosion was confirmed by detection of hemopericardium based on the density and Hounsfield CT number of the pericardial fluid and retroaortic hematoma in the first patient. Echocardiography may not be enough to rule out cardiac erosion. CTA can reveal information about structures that are difficult to visualize with TEE such as erosion of an ASD closure device into adjacent structures and performing cardiac CTA and looking for the evidence of cardiac erosion are essential [10].

The uncommon cause of PE, post TCC of ASD is case reports of seroritis (pleuro-pericarditis) which the underlying stimulus is not clear, but one hypothesis is the reaction to Nickle and the materials of the device; it seems to respond well to anti-inflammatory agents with low recurrence rate. In some patients, the underlying advanced RV failure leads to persistent PE even after ASD closure who have a very poor prognosis. In fact, this group of patients with advanced right ventricular failure which is somewhat irreversible develop pericardial effusion after ASD closure due to RV failure and require treatment for right ventricular failure and they have increased risk for atrial and ventricular arrhythmia. finally, in some cases, no reasonable source of PE could be found and extensive work-up for acute, sub-acute, and chronic pericarditis should be performed.

## Conclusion

Our experience in the management of these three patients highlighted that recognizing the different causes of pre-procedure and post-procedure pericardial effusion using diagnostic techniques such as echocardiography as well as another imaging modality such as cardiac CTA could be very useful and important for proper diagnosis, and management of worsening pre-existing PE in patients after ASD device closure. Cardiac CTA is a reliable diagnostic method for the detection of device erosion or cardiac perforation in patients who underwent transcatheter ASD closure with new onset or worsening of pre-existing PE.

## Data Availability

Data sharing is not applicable to this article as no datasets were generated or analyzed during the current study.

## Abbreviations

ASD: Atrial septal defect
TCC: Trans catheter closure
ESRD: End-stage renal disease
TEE: Transesophageal echocardiography
LV: Left ventricle
RV: Right ventricle
TR: Tricuspid regurgitation
TRG: Tricuspid regurgitant gradient
PAH: Pulmonary arterial hypertension
LVEDP: Left ventricular end-diastolic pressure
PVR: Pulmonary vascular resistance
LA: Left atrium
CTA: CT angiography
PE: Pericardial effusion
